# Vulnerability and Immunogenic Cell Death under Microwave Ablation for Synergistic Treatment of Hepatocellular Carcinoma

**DOI:** 10.34133/bmr.0421

**Published:** 2026-10-08

**Authors:** Zide Chen, Hongwei Xu, Yufu Peng, Mengni Zhu, Linqing Huang, Feng Fang, Kefei Chen

**Affiliations:** ^1^ Department of Interventional Radiology, Guangdong Provincial People’s Hospital, Guangdong Academy of Medical Sciences, Southern Medical University, Guangdong 510080, China.; ^2^ School of Medicine, South China University of Technology, Guangdong 510006, China.; ^3^ Division of Liver Surgery, Department of General Surgery, West China Hospital, Sichuan University, Chengdu 610041, China.; ^4^ Shanghai Key Laboratory of Magnetic Resonance, School of Physics and Electronic Science, East China Normal University, Shanghai 200241, China.; ^5^ The Third Clinical Medical College, Nanjing University of Chinese Medicine, Nanjing 210023, China.; ^6^ Department of Hepatobiliary Cancer, Liver Cancer Research Center, Tianjin Medical University Cancer Institute and Hospital, Tianjin 300060, China.

## Abstract

Microwave ablation (MWA) holds great promise for hepatocellular carcinoma but is often limited by incomplete tumor eradication and recurrence. Here, we report a tumor-killing nanoimmunoreactor driven by endogenous immune cues that synergizes with MWA to eliminate residual tumors and activate systemic antitumor immunity. The 17-AAG–MnO_2_PEG nanoimmunoreactor consists of a self-responsive PEGylated MnO_2_ platform loaded with the HSP90 inhibitor 17-allylamino-17-demethoxygeldanamycin (17-AAG), which is designed to respond to the oxidative tumor microenvironment. 17-AAG disrupts tumor cell thermotolerance, enhancing sensitivity to microwave-induced thermal stress, thereby improving ablation efficacy and inducing immunogenic cell death. PEGylation facilitates tumor-targeted accumulation, while the degradable MnO_2_ responds to hydrogen peroxide (H_2_O_2_) and tumor debris generated after MWA, enabling sustained local drug release. The combination treatment promotes dendritic cell maturation and T cell infiltration, effectively suppresses residual tumor regrowth, reduces the risk of metastasis, and establishes durable immune memory. By leveraging post-ablation tumor debris and microenvironmental cues, this strategy couples local tumor ablation with systemic antitumor immunity, providing a mechanistically distinct paradigm for durable cancer therapy.

## Introduction

Microwave ablation (MWA) has emerged as a minimally invasive approach for localized tumor eradication, utilizing high-frequency electromagnetic waves to induce rapid heating and irreversible cell damage [1,2]. Clinically, MWA demonstrates efficacy in shrinking tumors and preserving surrounding healthy tissue. However, a major limitation remains: Sublethal thermal injury frequently leaves behind residual tumor cells, which can survive due to adaptive stress responses and lead to recurrence and metastasis [3]. At the molecular level, these surviving cells up-regulate heat shock proteins HSP70 and HSP90, stabilizing denatured proteins and maintaining proteostasis under thermal stress [4–7]. In parallel, the oxidative and immunosuppressive tumor microenvironment (TME), characterized by elevated reactive oxygen species (ROS), hypoxia, and acidic pH, blunts local immune activation and prevents efficient antigen presentation [8]. While MWA can induce release of tumor-associated antigens and damage-associated molecular patterns (DAMPs), this alone is often insufficient to trigger robust dendritic cell (DC) maturation or cytotoxic T lymphocyte (CTL) responses, limiting systemic antitumor immunity [9–12]. These observations highlight the need for strategies that transform local ablation-induced tumor cell death into a potent, systemic immune response.

To address the limitations of MWA, we hypothesized that residual tumor debris could serve as an endogenous immune adjuvant when combined with a suitably designed therapeutic formulation. By exploiting the oxidative stress-rich TME and the immunogenic potential of ablation-induced cell death, such a strategy could enhance DC activation, promote antigen presentation, and prime CD4^+^ helper and CD8^+^ cytotoxic T cells to recognize and eliminate residual tumor cells [13–16]. Activation of innate immune pathways, including stimulator of interferon genes (STING) and nuclear factor κB (NF-κB), in response to debris-associated DAMPs and ROS can induce type I interferon signaling and pro-inflammatory cytokine production [interleukin-2 (IL-2), IL-6, tumor necrosis factor-α (TNF-α), interferon-γ (IFN-γ)], establishing a systemic antitumor milieu [17,18]. Furthermore, combining physical ablation with biochemical modulation could overcome tumor thermotolerance mediated by HSPs, thereby not only eliminating surviving tumor cells locally but also promoting durable immune memory [19–24]. This concept forms the basis for a debris-fueled, self-amplifying immunotherapy that synergizes with MWA to achieve both local tumor control and systemic antitumor immunity [25,26].

Unlike conventional drug delivery nanoplatforms that primarily function as passive or active carriers for therapeutic agents, our nanoimmunoreactor integrates 3 core functions—drug delivery, microenvironmental responsiveness, and immune activation [27,28]. Specifically, it responds to endogenous TME signals (e.g., ROS, hypoxia, and tumor debris generated post-ablation) to trigger in situ drug release while simultaneously inducing immunogenic cell death (ICD), promoting antigen presentation, and priming T cell responses. In addition, it synergizes with MWA by utilizing ablation-generated tumor debris as an endogenous “fuel” to amplify the immune response, a feature that fundamentally distinguishes it from conventional MWA or immunotherapy alone.

In this work, we designed a self-responsive PEGylated MnO_2_ nanosystem incorporating the HSP90 inhibitor 17-allylamino-17-demethoxygeldanamycin (17-AAG) to address the twin challenges of incomplete ablation and insufficient immune activation associated with MWA. We hypothesize that ablation-induced tumor components, together with the oxidative TME, can be exploited as endogenous triggers to activate the nanosystem in situ, enabling coordinated modulation of redox balance and cellular stress responses. Specifically, MnO_2_-mediated redox reprogramming is expected to reshape the post-ablation tumor milieu, while pharmacological inhibition of heat shock proteins is anticipated to disrupt tumor thermotolerance and enhance susceptibility to thermal injury. Therefore, the objective of this study is to establish a mechanism-guided nanochemical strategy that couples physical tumor ablation with biochemical stress modulation, with the ultimate aim of converting local ablation into a programmable and immunologically active therapeutic process (Fig. 1).

**Fig. 1.**
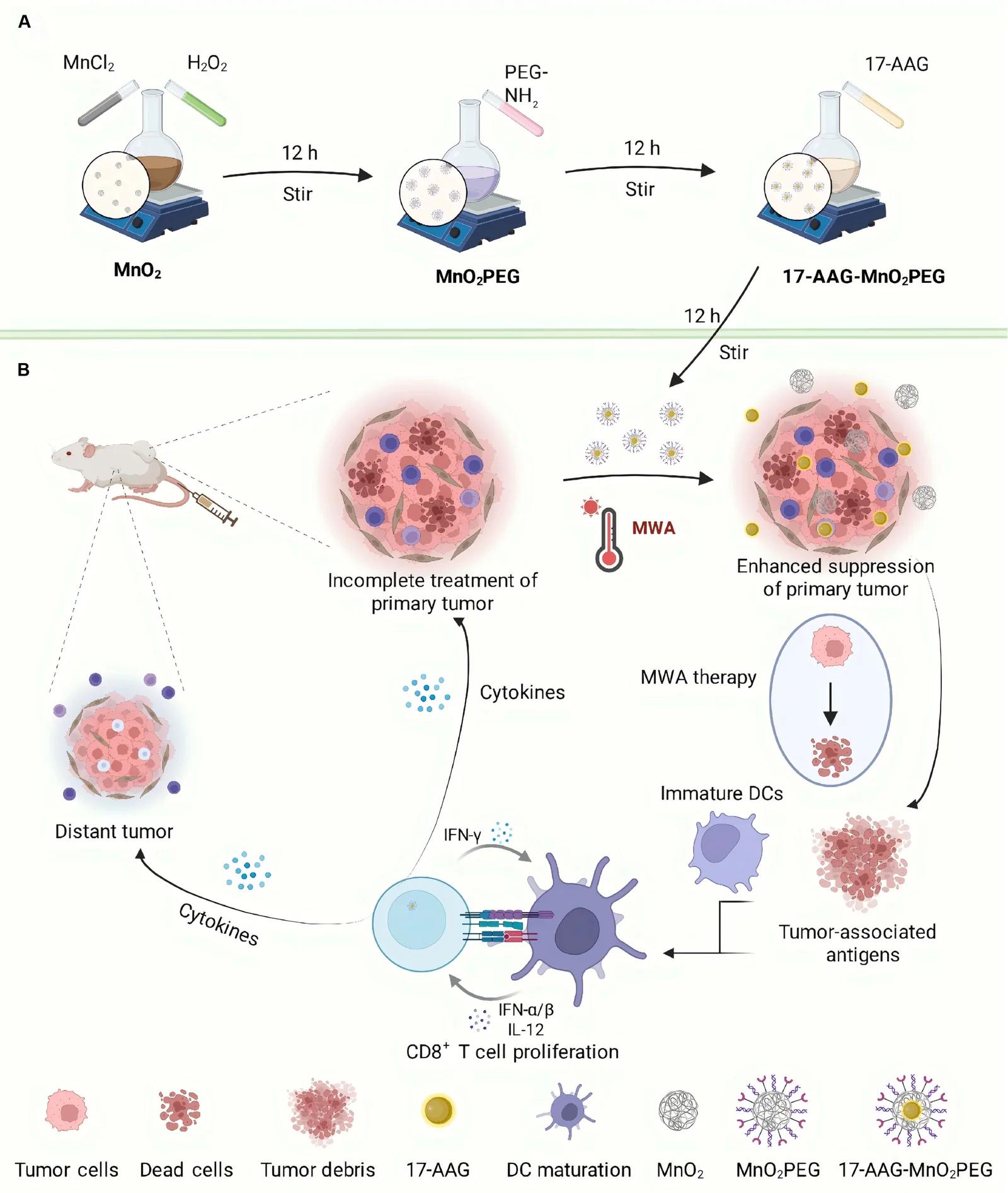
Design and mechanism of 17-AAG–MnO_2_PEG nanoreactors combined with MWA. (A) Preparation of PEGylated MnO_2_ nanoparticles loaded with 17-AAG. (B) MWA combined with HSP inhibition disrupts tumor thermotolerance, generates tumor debris, and induces ICD, thereby suppressing residual tumors and may contribute to the control of distant tumor progression through systemic immune activation.

## Materials and Methods

### Materials

Manganese(II) chloride tetrahydrate (MnCl_2_·4H_2_O, ≥99%), hydrogen peroxide (H_2_O_2_, 30 wt %), methanol (CH₃OH), ethanol (EtOH), dimethyl sulfoxide (DMSO), indocyanine green (ICG), and tribromoethanol were purchased from Aladdin Reagent Co. Ltd. (Shanghai, China). Polyethylene glycol amine (PEG-NH_2_, molecular weight ~5 kDa), 17-AAG (≥98%), Cell Counting Kit-8 (CCK-8), calcein-AM/propidium iodide (PI) live–dead staining kit, and Annexin V–fluorescein isothiocyanate (FITC)/PI apoptosis detection kit were obtained from MedChemExpress (Shanghai, China). RPMI 1640 medium, Dulbecco’s modified Eagle’s medium (DMEM), fetal bovine serum (FBS), and phosphate-buffered saline (PBS) were purchased from Gibco (USA). FITC-conjugated anti-CD80 and phycoerythrin (PE)-conjugated anti-CD86 antibodies were obtained from BioLegend (USA). Enzyme-linked immunosorbent assay (ELISA) kits for IL-6 and TNF-α were purchased from MultiSciences Biotech (Hangzhou, China). Trypsin-EDTA (0.25%) and paraformaldehyde were purchased from Sigma-Aldrich (USA). Dialysis membranes [molecular weight cutoff (MWCO): 3.5 kDa] were purchased from Spectrum Laboratories (USA). All chemicals were of analytical grade and used as received.

### Synthesis of MnO_2_PEG

A mixed solution of 3 wt % H_2_O_2_ was rapidly added to 10 ml of 0.3 M MnCl_2_ solution. After mixing, the resulting dark brown suspension was stirred vigorously in air at room temperature overnight. Next, the resulting lamellae of MnO_2_ were centrifuged at 2,000 rpm for 10 min, followed by several washes with large amounts of distilled water and methanol (CH₃OH). After washing, the MnO_2_ was dried at 60 °C and stored for later use. In a further modification step, 10 mg of MnO_2_ was dispersed in water, 10 mg of PEG-NH_2_ was added, and the reaction was carried out in 10 ml of water overnight. At the end of the reaction, MnO_2_PEG was obtained by centrifugal separation and washing.

### Synthesis of 17-AAG–MnO_2_PEG

The reaction was carried out by taking 3 ml of MnO_2_PEG aqueous dispersion at a concentration of 2.5 mg/ml, adding 1 ml of EtOH containing 3 mg/ml 17-AAG, and stirring the reaction overnight at room temperature. After completion of the reaction, the precipitate was collected by centrifugation and washed several times to obtain the 17-AAG–MnO_2_PEG complex. The drug loading content (DLC) and encapsulation efficiency (EE) were calculated by measuring the ultraviolet–visible (UV–Vis) absorbance of the supernatant after the reaction, with detailed calculation procedures provided in the Supplementary Materials.

### Materials characterization

The morphology and structure of MnO_2_PEG and 17-AAG–MnO_2_PEG were characterized by transmission electron microscopy (TEM, JEOL, Japan) and scanning electron microscopy (SEM, Hitachi, Japan). Hydrodynamic diameter and zeta potential were measured using a Zetasizer Nano ZS (Malvern Instruments, UK) at 25 °C. The ROS-scavenging capacity of MnO_2_PEG was evaluated using a Fenton reaction system (Fe^2+^/H_2_O_2_) by monitoring UV–Vis absorption with a UV-2600 spectrophotometer (Shimadzu, Japan). Nanoparticle stability was assessed in 10% DMSO, 10% FBS, and aqueous solutions using dynamic light scattering (DLS) over time. MWA performance was measured by irradiating nanoparticle dispersions with 2.5-GHz MWA (5 W) and recording temperature changes with a thermocouple digital thermometer. Drug loading and EE were quantified by UV–Vis spectroscopy (Shimadzu, Japan) using a pre-established standard curve. In vitro drug release was performed using dialysis bags (MWCO: 3.5 kDa) in PBS with or without H_2_O_2_ under constant agitation at 37 °C, with periodic sampling for UV–Vis measurement.

### ROS-scavenging assay

The hydroxyl radical (·OH) scavenging ability of MnO_2_PEG was evaluated using a Fenton reaction system. Briefly, FeSO₄ (50 μM) and H_2_O_2_ (100 μM) were mixed in PBS to generate hydroxyl radicals. Sodium salicylate (5 mM) was added as a probe, which reacts with ·OH to form 2,3-dihydroxybenzoic acid, detectable by its characteristic absorbance at 510 nm. MnO_2_PEG at various concentrations (0 to 100 μg/ml) was then added to the reaction system, and the mixture was incubated at 37 °C for 30 min. The absorbance of 2,3-dihydroxybenzoic acid was measured using a UV–Vis spectrophotometer (UV-2600, Shimadzu, Japan). The hydroxyl radical scavenging efficiency (%) was calculated as:

$$\text{Scavenging efficiency}\;\left(\%\right)=\frac{A_{0}-A_{s}}{A_{0}}\times 100$$ (1)

where $A_{0}\;$and $A_{s}\;$represent the absorbance values of the control (without MnO_2_PEG) and the sample (with MnO_2_PEG), respectively.

### Drug loading and in vitro release

To prepare the 17-AAG-loaded MnO_2_PEG nanosystem, 3 ml of MnO_2_PEG aqueous dispersion (2.5 mg/ml) was mixed with a solution of 3 mg of 17-AAG dissolved in 1 ml of EtOH. The mixture was stirred at room temperature overnight to allow drug adsorption. The resulting suspension was centrifuged at 10,000 rpm for 10 min, and the supernatant was collected. The amount of unbound 17-AAG in the supernatant was quantified using UV–Vis spectroscopy (UV-2600, Shimadzu, Japan) to calculate the DLC and EE. The DLC was determined to be 14.7%, and the EE was 57.3%. For in vitro drug release, 17-AAG–MnO_2_PEG was placed in dialysis bags (MWCO: 3.5 kDa) and incubated in PBS (pH 7.4) containing 10 mM H_2_O_2_ at 37 °C under constant agitation, with or without 5-W microwave irradiation. At predetermined time points over 72 h, aliquots of the release medium were collected and replaced with fresh PBS. The concentration of released 17-AAG in the collected samples was determined by UV–Vis spectroscopy, and the cumulative release profile was calculated. All release experiments were performed in triplicate (*n* = 3), and data are presented as mean ± SD.

### Microwave heating performance measurement

Aqueous dispersions of MnO_2_PEG at concentrations of 0, 25, 100, and 200 μg/ml (1 ml each) were prepared and placed in an MWA device (2.5 GHz, 5 W). Temperature changes were recorded with a digital thermometer during microwave irradiation to generate temperature–time curves. For material characterization, the heating process was recorded for up to 600 s to evaluate thermal stability at different concentrations, while for cellular and animal experiments, a 2-min irradiation duration was applied.

### Cell viability assay

Human hepatocellular carcinoma cell lines HepG2 and SK-Hep-1, as well as murine hepatoma cell line Hepa1-6, were obtained from the Cell Bank of the Chinese Academy of Sciences (Shanghai, China). All cell lines were authenticated by short tandem repeat (STR) profiling and routinely tested negative for mycoplasma contamination prior to use. HepG2 and SK-Hep-1 cells were cultured in DMEM supplemented with 10% FBS and 1% penicillin–streptomycin, while Hepa1-6 cells were maintained in RPMI 1640 medium supplemented with 10% FBS under standard cell culture conditions (37 °C, 5% CO_2_ atmosphere). The cytotoxicity of MnO_2_PEG, 17-AAG, and 17-AAG–MnO_2_PEG was detected by CCK-8 assay. First, HepG2 and SK-Hep-1 hepatocellular carcinoma cells were inoculated into 96-well plates at a density of 1 × 10^4^ cells/well and cultured overnight. Next, different concentrations of MnO_2_PEG, 17-AAG, and 17-AAG–MnO_2_PEG were added, and incubation was continued for another 24 h. Then, the medium was replaced with serum-free medium containing 10% CCK-8 and further incubated for 1 h. Finally, the absorbance at 450 nm was measured by a microplate reader to calculate cell viability.

To evaluate the cancer selectivity of our nanoplatform, normal hepatocytes (LO2) were included as a control alongside hepatocellular carcinoma (HCC) cell lines (HepG2 and SK-Hep-1) for comparative cytotoxicity and cellular uptake assays. The results showed markedly lower cytotoxicity and weaker cellular uptake of 17-AAG–MnO_2_PEG in LO2 cells compared to HCC cell lines, supporting the tumor-selective profile of our nanoplatform.

### Intracellular ROS detection

To clarify the role of MnO_2_ in regulating ROS, intracellular ROS levels were assessed using the 2′,7′-dichlorodihydrofluorescein diacetate (DCFH-DA) probe. HepG2 and SK-Hep-1 cells were seeded in 12-well plates and treated according to the grouping protocol: NC (untreated), MWA alone (5 W, 2 min), 17-AAG + MWA (preincubated with 50 μg/ml free 17-AAG for 4 h followed by MWA exposure), MnO_2_PEG + MWA, and 17-AAG–MnO_2_PEG + MWA. After treatment, cells were incubated for 24 h, stained with 10 μM DCFH-DA for 30 min at 37 °C in the dark, washed twice with PBS, and immediately visualized by confocal fluorescence microscopy. Fluorescence intensity was quantified using ImageJ software.

### Identification of live and dead cells

HepG2 and SK-Hep-1 cells were inoculated into 24-well plates at 1 × 10^5^ cells per well and cultured overnight. The cells were divided into 4 groups: normal control (NC), MWA (5 W, 2 min), MnO_2_PEG + MWA (incubated with 50 μg/ml of MnO_2_PEG for 4 h, followed by exposure of cells to the MWA environment for 2 min), and 17-AAG–MnO_2_PEG + MWA (incubated with 50 μg/ml of 17-AAG–MnO_2_PEG incubation for 4 h, then cells were exposed to MWA environment for 2 min). After 1 h of incubation, the cells were stained with the calcein-AM/PI kit for 10 min, followed by washing with PBS. Finally, the staining results were observed using confocal fluorescence microscopy.

### Apoptosis assay

HepG2 and SK-Hep-1 cells were inoculated into 6-well plates at 5 × 10^5^ cells per well and cultured overnight. Cells were divided into 4 groups: normal control (NC), MWA (5 W, 2 min), MnO_2_PEG + MWA (incubated with 50 μg/ml of MnO_2_PEG for 4 h, followed by exposure of cells to the MWA environment for 2 min), and 17-AAG–MnO_2_PEG + MWA (incubated with 50 μg/ml of 17-AAG–MnO_2_PEG incubation for 4 h, then cells were exposed to the MWA environment for 2 min). After collecting and washing the cells, the cells were resuspended using 500 μl of buffer, and staining was performed by adding 5 μl each of PI and Annexin-V FITC for 10 min. Finally, apoptosis was detected by flow cytometry.

### Cell uptake staining

To assess the uptake of R6G@MnO_2_PEG and R6G@17-AAG–MnO_2_PEG by HepG2 and SK-Hep-1 cells, the cells were treated with 50 μg/ml of R6G@MnO_2_PEG and R6G@17-AAG–MnO_2_PEG, respectively, and irradiated with MWA according to the grouping. After 3 h of incubation, the cells were washed twice with prewarmed PBS, and the washed cells were subsequently collected. Finally, the intracellular fluorescence intensity of R6G@MnO_2_PEG and R6G@17-AAG–MnO_2_PEG was quantitatively analyzed using laser confocal microscopy.

### Western blot

HepG2 and SK-Hep-1 cells were inoculated into 6-well plates at 5 × 10^5^ cells per well and cultured overnight. Cell grouping and treatment were the same as the cell live–dead staining assay. At the end of incubation, cells were collected and total cellular proteins were extracted. Protein expression levels of HSP70 and HSP90 were detected using protein immunoblotting.

### Isolation of primary BMDCs

Immature bone marrow-derived DCs (BMDCs) were isolated from mice. The obtained immature BMDCs were inoculated in 24-well plates. Cells were treated with NC, MnO₂PEG, MnO₂PEG + MWA, 17-AAG–MnO₂PEG, and 17-AAG–MnO₂PEG + MWA and irradiated with microwave according to the grouping. After 3 h of incubation, the cells were washed twice with prewarmed PBS and collected. Then, HepG2 cells and their medium were co-incubated with BMDCs for 24 h. At the end of the incubation, the HepG2 cells in the upper chamber were discarded, and the suspended cells and sparsely adherent growing cells were collected. The expression levels of IL-6 and TNF-α in the cell supernatants were detected by centrifugation followed by ELISA. Subsequently, the cells were resuspended in tubes and stained with FITC-CD80 and PE-CD86 antibodies to avoid light, and finally detected by flow cytometry.

### Immunofluorescence staining of calreticulin

For calreticulin (CRT) exposure detection, HepG2 and SK-Hep-1 cells were seeded in 12-well plates (1 × 10^5^ cells/well) overnight and divided into 5 groups: NC (untreated), MWA alone (5 W, 2 min), 17-AAG + MWA (preincubated with 50 μg/ml free 17-AAG for 4 h followed by MWA exposure), MnO_2_PEG + MWA (preincubated with 50 μg/ml MnO_2_PEG for 4 h followed by MWA exposure), and 17-AAG–MnO_2_PEG + MWA (preincubated with 50 μg/ml 17-AAG–MnO_2_PEG for 4 h followed by MWA exposure). After treatment, cells were fixed with 4% paraformaldehyde for 15 min, blocked with 5% goat serum for 30 min, and incubated with anti-CRT primary antibody at 4 °C overnight, followed by fluorescence-conjugated secondary antibody incubation. Nuclei were counterstained with 4′,6-diamidino-2-phenylindole (DAPI), and images were acquired by confocal fluorescence microscopy.

### Screening for safe concentrations

All animal experiments were conducted in accordance with the guidelines of the Animal Care and Use Committee and were approved by the West China Hospital of Sichuan University (approval no. 20250221007). Male BALB/c nude mice (6 to 8 weeks old, 18 to 22 g) and male BALB/c mice (6 to 8 weeks old) were purchased from Shanghai SLAC Laboratory Animal Co. Ltd. (Shanghai, China) and maintained under specific pathogen-free (SPF) conditions. All animals were housed in a controlled environment with a 12-h light/dark cycle, temperature of 22 ± 2 °C, and relative humidity of 50% to 60%, with free access to standard laboratory chow and water. Twelve BALB/c mice were randomly divided into 3 groups: the PBS group, the MnO_2_PEG group, and the 17-AAG–MnO_2_PEG group. After 24 h of drug administration by tail vein injection of different concentrations, each mouse was anesthetized by intraperitoneal injection of 0.25 ml of tribromoethanol, followed by decapitation. Finally, the survival of the mice and the appearance of the liver and kidney were recorded.

For long-term toxicity assessment of 17-AAG–MnO_2_PEG, additional experiments were performed to evaluate hemolysis and key biochemical parameters. For the hemolysis assay, fresh mouse red blood cells were incubated with 17-AAG–MnO_2_PEG at various concentrations (0, 25, 50, 100, and 200 μg/ml) for 4 h at 37 °C, and the hemolysis rate was calculated. For biochemical analysis, blood samples were collected at 28 d post-injection from BALB/c mice following intravenous administration of 17-AAG–MnO_2_PEG (5 mg/kg). Serum biochemical parameters including alanine aminotransferase (ALT), aspartate aminotransferase (AST), alkaline phosphatase (ALP), blood urea nitrogen (BUN), and creatinine (CREA) were measured using an automatic biochemical analyzer.

### Establishment of the in vivo model and biodistribution

Logarithmic phase HepG2 cells (2 × 10^6^) were subcutaneously injected into the left axilla of male Balb/c nude mice. After injection, tumor growth was observed every 2 d and tumor volume was measured using the following formula:

$$\text{Tumor volume}\;\left({\mathrm{mm}}^{3}\right)=\text{length}\times {\left(\text{width}\right)}^{2}\times 0.5$$ (2)

After the model was established, mice with similar tumor sizes were randomly divided into 3 groups and injected with PBS, ICG, and 17-AAG–MnO_2_PEG-ICG nanomedicines via tail vein, respectively. Then, in vivo imaging was performed to detect the drug metabolism and the targeting effect of the nanomedicine at different time points. In addition, various organs of mice were taken after 72 h for tissue imaging to study the drug metabolism and the targeting effect of nanomedicines.

### In vivo therapeutic assay

The established mouse models were used for the experiment, and mice with tumors of comparable size and growth rate were randomly assigned into 5 groups. The groups were administered 100 μl of PBS, MnO_2_PEG, 17-AAG, 17-AAG–MnO_2_PEG, or 17-AAG–MnO_2_PEG + MWA nanodrug via tail vein injection, at a dosage of 5 mg/kg. Tumor inhibition rates and survival rates of the mice were measured based on the different treatments. At the end of the treatment period, tumors were collected for analysis, and hematoxylin and eosin (H&E) staining, CD31, terminal deoxynucleotidyl transferase–mediated deoxyuridine triphosphate nick end labeling (TUNEL), and Ki67 immunofluorescence staining were performed to evaluate the therapeutic effects.

To investigate the distribution, accumulation, and metabolism of Mn ions, a semiquantitative analysis was performed using a manganese superoxide dismutase (Mn-SOD) activity assay in tumors and major organs (heart, liver, spleen, lung, and kidney) at 72 h post-injection. Tissue samples were homogenized, and Mn-SOD activity was measured using a commercial kit according to the manufacturer’s instructions, with results expressed as U/mg protein. This assay indirectly reflects bioavailable Mn levels in tissues, as Mn is an essential cofactor for Mn-SOD activity.

### Establishment of the animal model and evaluation of its immunomodulatory effects

Logarithmic phase Hepa1-6 cells (2 × 10^6^) were subcutaneously injected into the left axilla of male Balb/c nude mice. Tumor growth was monitored every 2 d, and tumor volume was measured. When the subcutaneous tumor volume reached 100 mm^3^, mice were administered 100 μl of PBS, MnO_2_PEG, 17-AAG, 17-AAG–MnO_2_PEG, or 17-AAG–MnO_2_PEG + MWA nanodrug via tail vein injection, at a dosage of 5 mg/kg. Immune responses in the Balb/c nude mice were evaluated at the end of the treatment period.

To evaluate T cell activation in an immunocompetent background, splenic lymphocytes were isolated from C57BL/6 mice and cocultured with Hepa1-6 mouse hepatoma cells for 24 h under various treatments (PBS + MWA, MnO_2_PEG, 17-AAG, MnO_2_PEG + MWA, 17-AAG + MWA, 17-AAG–MnO_2_PEG, and 17-AAG–MnO_2_PEG + MWA). After co-incubation, cells were collected, washed, and Fc receptors were blocked. Two parallel staining panels were set up: CD4-PE with CD25-allophycocyanin (APC) for detecting CD4^+^ T cell activation, and CD8-PE with CD25-APC for detecting CD8^+^ T cell activation. Stained cells were analyzed by flow cytometry, with sequential gating for cell population, single cells, live cells, and CD3^+^ lymphocytes, followed by CD4/CD25 or CD8/CD25 quadrant analysis.

### Statistical significance

All data are presented as mean ± SD from at least 3 independent experiments (*n* = 3). Comparisons between 2 groups were assessed via 2-tailed Student’s *t* tests. For multiple-group comparisons, one-way analysis of variance (ANOVA) followed by Tukey’s multiple comparisons test was employed. ns = not significant, *P < 0.05, **P < 0.01, ***P < 0.001, ****P < 0.0001. All error bars in figures represent standard deviation (SD) from 3 independent replicates.

## Results

### Characterization and functional evaluation of 17-AAG–MnO_2_PEG nanosystem

The 17-AAG–MnO_2_PEG nanosystem was synthesized as a multifunctional platform for redox-responsive drug release and thermotolerance modulation in the TME. It was then systematically characterized. TEM and SEM images revealed that the MnO_2_PEG nanoparticles exhibited a crystalline structure with a particle size of approximately 250 nm (Fig. 2A and Fig. S1). UV–Vis measurements were further conducted on the core MnO_2_PEG nanoparticles (Fig. S2), confirming their morphology, size, and electronic structure. DLS analysis revealed hydrodynamic sizes of 171.6 nm for MnO_2_, 177.8 nm for MnO_2_PEG, and 275.2 nm for 17-AAG–MnO_2_PEG, with corresponding polydispersity index (PDI) values of 0.489, 0.507, and 0.480, respectively, indicating narrow size distribution and good dispersibility (Fig. 2B). Conjugation with 17-AAG produced the final 17-AAG–MnO_2_PEG nanosystem, which showed a slight increase in particle size, while the zeta potential remained essentially unchanged, indicating successful drug loading without markedly altering the surface charge (Fig. 2C). Quantification of residual free drug by UV–Vis revealed a 17-AAG DLC of 14.7% and an EE of 57.3% on MnO_2_PEG (Fig. S3).

**Fig. 2.**
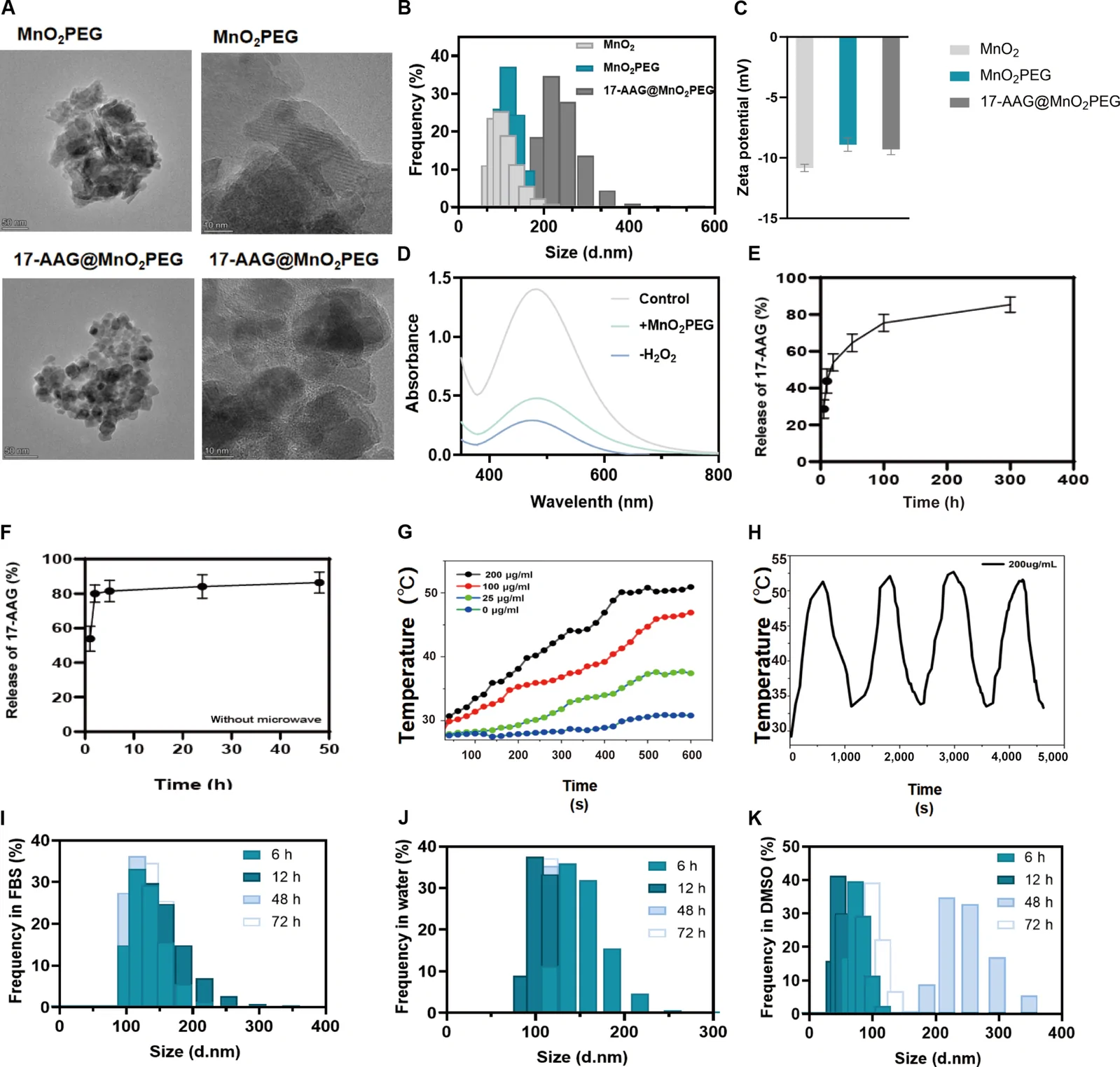
Characterization and functional evaluation of 17-AAG–MnO_2_PEG nanosystem. (A) TEM images of MnO_2_PEG and 17-AAG–MnO_2_PEG. (B and C) Particle size and zeta potential measurements of MnO_2_, MnO_2_PEG, and 17-AAG–MnO_2_PEG. (D) Assessment of ·OH scavenging ability using the Fenton reaction system. (E) In vitro cumulative release profile of 17-AAG from 17-AAG–MnO_2_PEG in PBS (pH 7.4) containing 10 mM H_2_O_2_. (F) Microwave-enhanced in vitro release profile of 17-AAG from 17-AAG–MnO_2_PEG in PBS (pH 7.4) containing 10 mM H_2_O_2_. (G and H) MWA performance and thermal stability of 17-AAG–MnO_2_PEG under 2.5 GHz. (I to K) Stability evaluation of 17-AAG–MnO_2_PEG in different solvent environments.

The drug release behavior of 17-AAG–MnO_2_PEG was subsequently evaluated using a dialysis method. The nanoparticles were sealed in dialysis bags (MWCO: 3.5 kDa) and immersed in PBS (pH 7.4) containing 10 mM H_2_O_2_ to simulate the oxidative TME. UV–Vis analysis demonstrated that 17-AAG–MnO_2_PEG exhibited a gradual and sustained release profile, with approximately 13% of the loaded 17-AAG released within the first 24 h and around 40% released after 80 h (Fig. 2E).

The effect of microwave irradiation on drug release was further investigated. Under the same conditions (PBS containing 10 mM H_2_O_2_), microwave irradiation markedly accelerated the release of 17-AAG compared with the non-irradiated group, resulting in a substantially higher cumulative release throughout the experimental period (Fig. 2F). These findings indicate that MWA not only promotes the degradation of MnO_2_PEG but also facilitates the on-demand release of 17-AAG at the ablation site, thereby enabling localized and controlled drug delivery to enhance the therapeutic efficacy of MWA [2].

Encouraged by the microwave-responsive drug release behavior, we next evaluated the heating performance of MnO_2_PEG under microwave irradiation to verify its suitability for thermosensitization-enhanced MWA therapy. As shown in Fig. 2G, MnO_2_PEG dispersions exhibited concentration-dependent heating under microwave irradiation, with the 200 μg/ml group reaching temperatures above 50 °C, which is sufficient for effective tumor ablation. In contrast, the 100 and 25 μg/ml groups showed moderate temperature increases, whereas the control group (0 μg/ml) exhibited negligible heating. MWA stability was confirmed over repeated heating/cooling cycles (Fig. 2H). To simulate the drug delivery microenvironment, we evaluated the stability of 17-AAG–MnO_2_PEG in 10% DMSO, 10% FBS, and aqueous solutions. The nanocomposite showed instability in 10% DMSO with noticeable particle size changes, which may be attributed to the disruption of the steric stabilization provided by the PEG shell and alterations in the dielectric constant of the solvent, thereby weakening the electrostatic repulsion between colloidal particles and leading to a certain degree of aggregation, while maintaining considerable stability in 10% FBS and aqueous solutions with minimal size variation (Fig. 2I to K). Collectively, these results demonstrate that 17-AAG–MnO_2_PEG integrates redox-responsive drug release with effective MWA performance. MWA-induced hyperthermia and oxidative stress accelerate MnO_2_ decomposition and promote in situ release of 17-AAG in residual tumors. The released 17-AAG suppresses heat shock protein-mediated thermotolerance, thereby sensitizing tumor cells to thermal injury, while MnO_2_-mediated redox modulation further amplifies cellular damage and promotes ICD. This synergy establishes a foundation for in vivo validation of enhanced ablation efficacy and immune activation.

### Enhanced cellular uptake and MWA-sensitized cytotoxicity of 17-AAG–MnO_2_PEG

Building on the physicochemical characterization and the demonstrated redox-triggered release behavior of 17-AAG–MnO_2_PEG, we next investigated whether these properties could be effectively translated into enhanced cellular internalization, thermotolerance modulation, and MWA-sensitized cytotoxicity at the cellular level. The cytotoxic effects of MnO_2_PEG, 17-AAG, and 17-AAG–MnO_2_PEG on HepG2 and SK-Hep-1 cells were assessed by cell viability assays (Fig. 3A). The results showed that both MnO_2_PEG and 17-AAG–MnO_2_PEG markedly reduced the viability of HepG2 and SK-Hep-1 cells in a concentration-dependent manner. Moreover, modification of 17-AAG did not markedly increase the cytotoxicity of 17-AAG–MnO_2_PEG. In addition, MWA-induced thermal therapy further improved the treatment effect with a significant decrease in cell viability compared to the untreated and control groups. By using rhodamine B (RG6) fluorescently labeled nanocomposites, cellular uptake of the nanocomposites was visualized using confocal microscopy (Fig. 3B). Red fluorescent signals were observed in HepG2 and SK-Hep-1 cells treated with MnO_2_PEG and 17-AAG–MnO_2_PEG, confirming the successful internalization of the nanocomposites. The 17-AAG–MnO_2_PEG-treated cells showed stronger fluorescence intensity compared to the MnO_2_PEG group, indicating enhanced cellular uptake. Quantitative analysis of cellular uptake based on fluorescence intensity (Fig. 3C) showed that the 17-AAG–MnO_2_PEG group showed a significant increase in fluorescence intensity in both cell lines, suggesting that the 17-AAG modification contributes to a more efficient nanoparticle internalization.

**Fig. 3.**
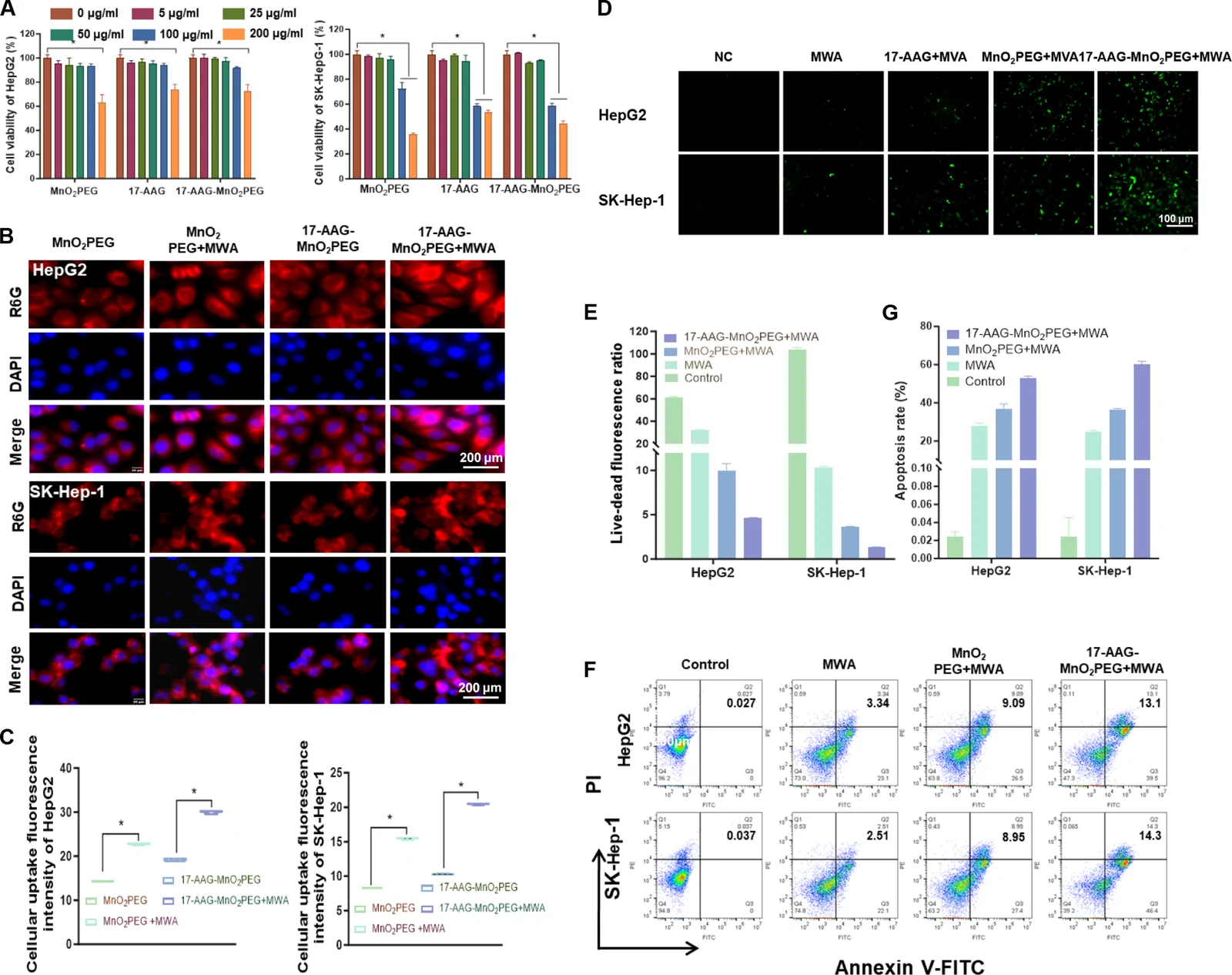
In vitro evaluation of 17-AAG–MnO_2_PEG in HepG2 and SK-Hep-1 cells. (A) Cell viability assays to assess cytotoxicity of MnO_2_PEG, 17-AAG, and 17-AAG–MnO_2_PEG. (B) Confocal microscopy imaging of cellular uptake using Rhodamine B-labeled nanocomposites. (C) Quantitative analysis of fluorescence intensity from cellular uptake. (D and E) Live/dead staining of HepG2 and SK-Hep-1 cells and corresponding quantitative fluorescence analysis. (F and G) Flow cytometry analysis of apoptosis after different treatments and corresponding quantitative fluorescence analysis. *n* = 3, *P* < 0.05.

As shown in Fig. S4A and B, the cytotoxicity of 17-AAG–MnO_2_PEG against LO2 cells was markedly lower than that against HepG2 and SK-Hep-1 cells, while cellular uptake in LO2 cells was also markedly weaker, supporting the tumor-selective profile of our nanoplatform. Quantitative analysis of cellular uptake (Fig. S4C to E) showed that fluorescence intensity in LO2 normal hepatocytes was markedly lower than that in HepG2 and SK-Hep-1 hepatocellular carcinoma cells, indicating the tumor-selective uptake profile of this nanoplatform. In both HCC cell lines, the 17-AAG–MnO_2_PEG + MWA group exhibited markedly higher fluorescence intensity than the other control groups, confirming that 17-AAG modification combined with MWA synergistically enhances the cellular internalization efficiency of the nanocomposites.

In addition, the fluorescence intensity of the MnO_2_PEG and 17-AAG–MnO_2_PEG groups further increased upon MWA, indicating enhanced cellular uptake. This effect is likely attributed to the transient increase in membrane fluidity and permeability induced by local MWA, which facilitates nanoparticle internalization through thermally activated endocytic pathway. Compared with MnO_2_PEG + MWA and 17-AAG + MWA, 17-AAG–MnO_2_PEG + MWA-treated HepG2 and SK-Hep-1 cells showed a markedly higher proportion of dead cells, as confirmed by live/dead staining and quantitative fluorescence analysis (Fig. 3D and E). Flow cytometry further revealed that this group exhibited the highest apoptotic rates among all treatments (Fig. 3F and G). These results indicate a synergistic effect: MWA provides thermal stress, while 17-AAG-mediated HSP inhibition reduces cellular thermotolerance and MnO_2_-triggered redox modulation enhances oxidative stress, together amplifying tumor cell death far beyond the effect of either MWA or nanoreactor treatment alone.

### MWA-mediated HSP regulation and ICD-driven DC activation by 17-AAG–MnO_2_PEG

To investigate the mechanism by which 17-AAG–MnO_2_PEG enhances MWA-induced tumor cell death, we first examined the expression of heat shock proteins HSP70 and HSP90 in HepG2 and SK-Hep-1 cells. Heat shock proteins are key mediators of cellular thermotolerance, and their down-regulation can sensitize tumor cells to thermal stress. The results showed that MnO_2_PEG + MWA treatment markedly up-regulated HSP70 and HSP90 expression, whereas in the 17-AAG–MnO_2_PEG + MWA group, HSP70 and HSP90 levels were markedly reduced, indicating that 17-AAG effectively suppresses heat shock protein expression and enhances tumor cell sensitivity to MWA-induced hyperthermia (Fig. 4A and B).

**Fig. 4.**
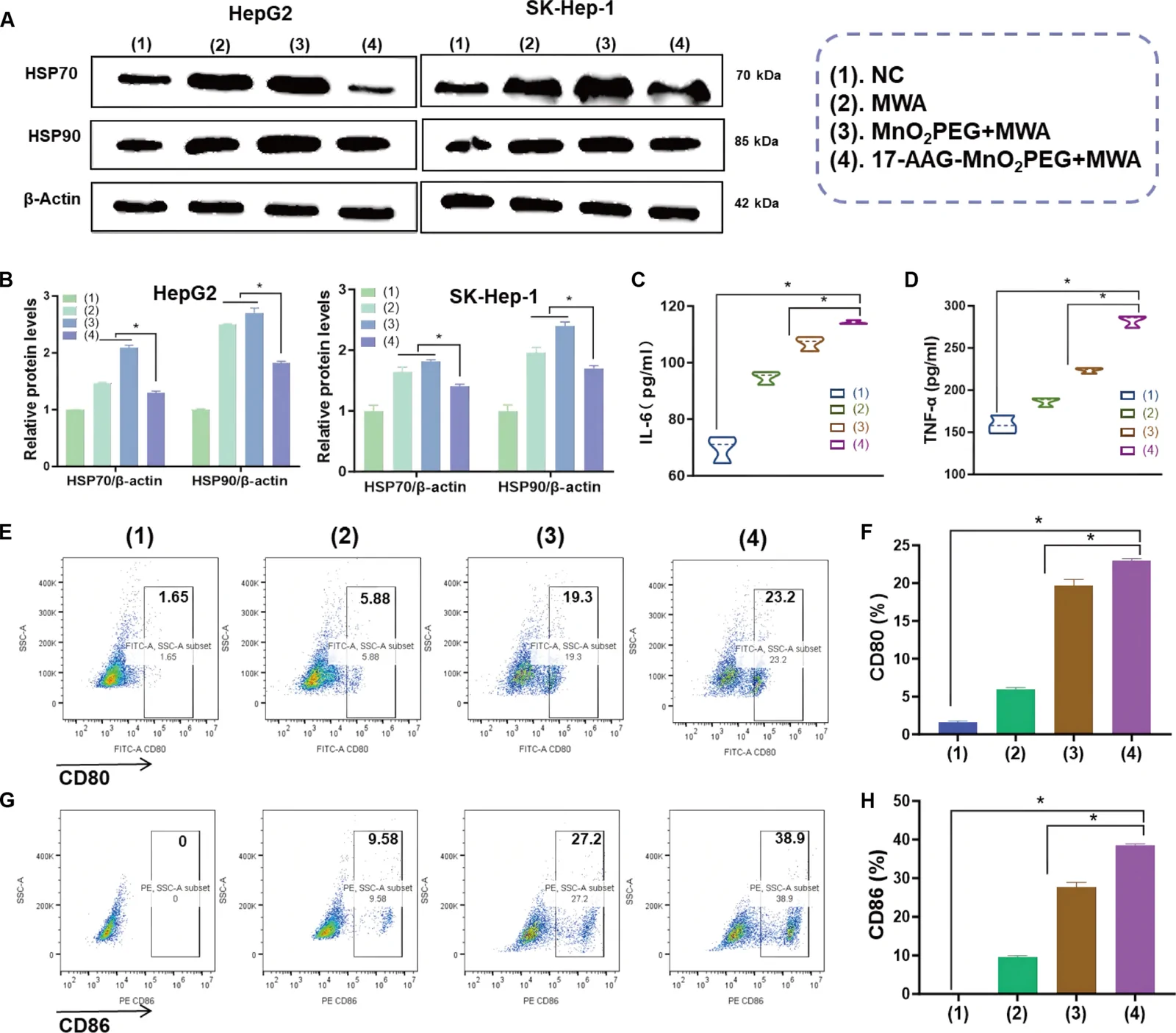
Mechanistic exploration of MWA sensitization and immune activation. (A) Western blot image of HSP70 and HSP90 expression in HepG2 and SK-Hep-1 cells after different treatments. (B) Relative protein expression levels of HSP70 and HSP90 in HepG2 and SK-Hep-1 cells (*n* = 3, *P* < 0.05). (C) ELISA quantitative analysis of IL-6 in different treatments. (D) ELISA quantitative analysis of TNF-α in different treatments (*n* = 3, *P < 0.05). (E) Quantitative analysis of CD80 fluorescence from different groups. (F) Quantitative analysis of CD80 expression levels (*n* = 3, *P < 0.05). (G) Quantitative analysis of CD86 fluorescence from different groups. (H) Quantitative analysis of CD86 expression levels (*n* = 3, *P* < 0.05).

Since the down-regulation of HSP70 and HSP90 enhances the sensitivity of tumor cells to hyperthermia, and hyperthermia-induced tumor cell death is often accompanied by the triggering of ICD-related molecular events, we further compared the effects of free 17-AAG versus 17-AAG–MnO_2_PEG combined with MWA treatment on cell surface CRT exposure and intracellular ROS generation in tumor cells, in order to verify whether the nanodelivery system could more effectively induce ICD and activate antitumor immunity. As shown in Fig. S5, HepG2 and SK-Hep-1 cells were treated with free 17-AAG (50 μg/ml, 4 h preincubation) followed by MWA (5 W, 2 min), and compared with the 17-AAG–MnO_2_PEG + MWA group. The results demonstrated that 17-AAG–MnO_2_PEG + MWA induced markedly higher CRT exposure on the cell surface and more potent intracellular ROS generation compared to free 17-AAG + MWA, indicating that nanoparticle encapsulation markedly enhances the therapeutic efficacy of 17-AAG.

We further evaluated ICD-mediated immune activation. HepG2 cells were selected as representative tumor cells and cocultured with immature BMDCs isolated from mice. The results demonstrated that HepG2 cells treated with MnO_2_PEG + MWA or 17-AAG–MnO_2_PEG + MWA markedly promoted BMDC maturation, with markedly increased expression of the costimulatory molecules CD80 and CD86. ELISA analysis of the culture supernatant showed markedly elevated levels of the pro-inflammatory cytokines IL-6 and TNF-α, with the 17-AAG–MnO_2_PEG + MWA group exhibiting stronger immune activation compared to the MnO_2_PEG + MWA group (Fig. 4C and D).

Flow cytometry further confirmed BMDC activation, as 17-AAG–MnO_2_PEG + MWA treatment substantially increased the proportion of CD80^+^ and CD86^+^ DCs (Fig. 4E to H), indicating enhanced antigen-presenting capability. MWA combined with nanomaterial treatment can convert a subset of non-immunogenic tumor cells into ICD. In summary, the 17-AAG–MnO_2_PEG system not only markedly amplifies ICD-mediated immune stimulation but also provides mechanistic support for tumor debris-driven immune activation observed in vivo during MWA combination therapy, effectively linking local ablation to systemic antitumor immunity.

### In vivo tumor targeting and distribution of 17-AAG–MnO_2_PEG

The biosafety of the nanodrugs was systematically evaluated through a dose-escalation study to determine the maximum tolerated dose and the safe concentration for subsequent in vivo experiments (Table S1). Mice were intravenously administered MnO_2_PEG or 17-AAG–MnO_2_PEG at graded doses, and toxicity was assessed by survival rate, gross morphology of the liver and kidneys, and histopathological examination of major organs via H&E staining. Results indicated that at doses starting from 10 mg/kg, no significant alterations were observed in liver and kidney appearance, and tissue architecture remained intact without evident necrosis or inflammatory infiltration (Figs. S6 and S7). Considering the need for repeated administrations in later experiments, a conservative dose of 5 mg/kg was selected to ensure systemic biosafety while maintaining therapeutic feasibility.

To evaluate the biodistribution and tumor-targeting efficiency of 17-AAG–MnO_2_PEG in vivo, dynamic fluorescence imaging was conducted in tumor-bearing mice at various time points (Fig. 5A). Compared to the control and ICG-only groups, the 17-AAG–MnO_2_PEG group exhibited markedly enhanced fluorescence signals at the tumor sites. Quantitative analysis of fluorescence intensity further confirmed reaching a maximum intensity at 48 h post-injection. Notably, while the fluorescence signals in the ICG group diminished after 3 h, those in the 17-AAG–MnO_2_PEG group remained robust at the tumor sites even at 48 h. Fluorescence signals in the ICG group were negligible at tumor locations, underscoring the superior tumor-targeting capacity and prolonged retention of 17-AAG–MnO_2_PEG in vivo. Its pronounced tumor-targeting capability arises from multiple intrinsic advantages of the nanosystem. The optimal particle size allows passive accumulation through the enhanced permeability and retention (EPR) effect of tumor vasculature. PEGylation prolongs circulation time in the bloodstream, increasing the likelihood of tumor accumulation. Meanwhile, the high levels of ROS in the TME trigger MnO_2_ degradation and local release of 17-AAG, enabling tumor-specific responsiveness. In addition, MnO_2_-mediated modulation of the TME, such as the conversion of H_2_O_2_ to O_2_, further enhances local drug retention while minimizing nonspecific accumulation in healthy tissues. Collectively, these factors work synergistically to preferentially enrich 17-AAG–MnO_2_PEG in tumor tissues, substantially reducing systemic toxicity.

**Fig. 5.**
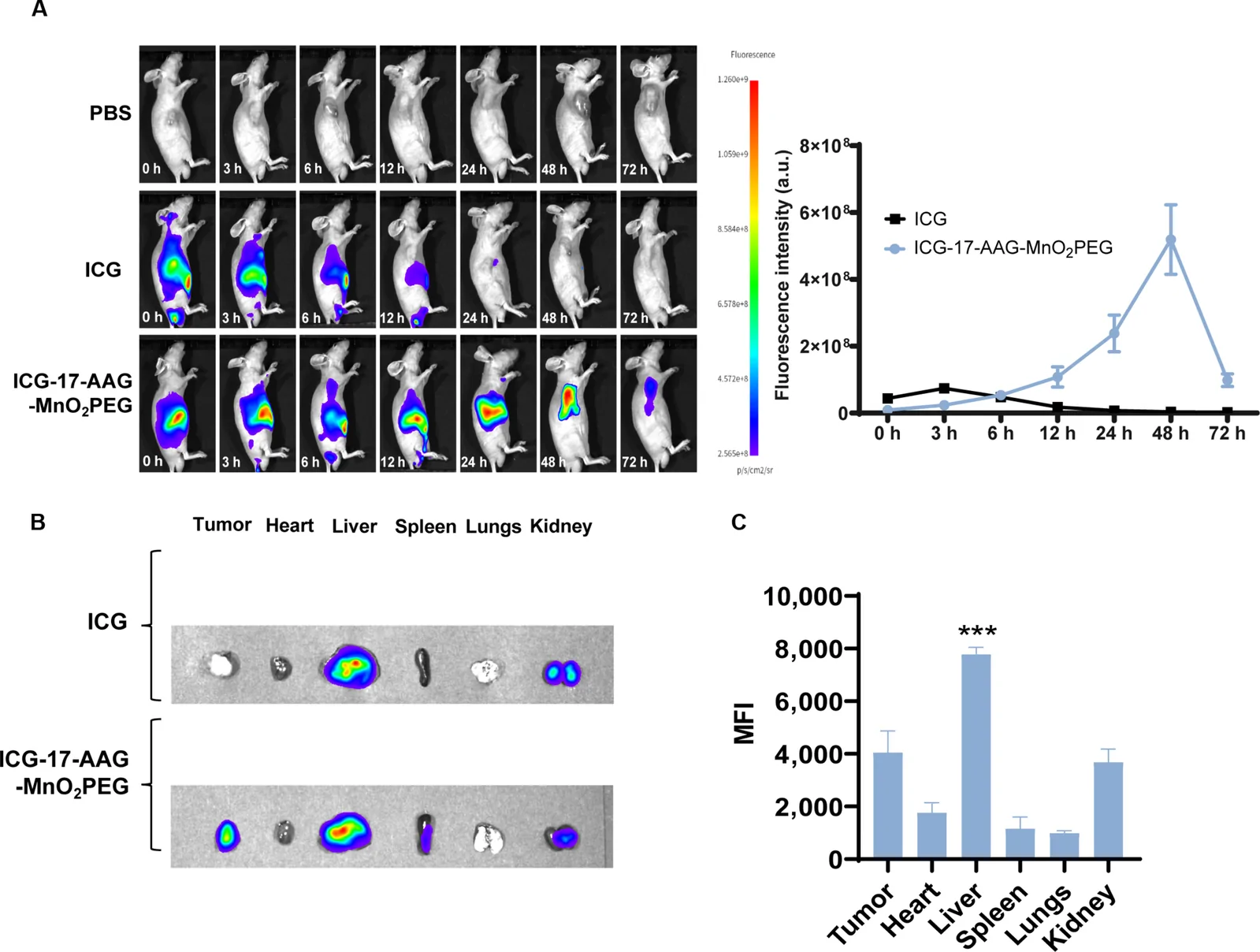
Distribution of 17-AAG–MnO_2_PEG in mice. (A) In vivo fluorescence imaging and quantitative analysis of mice after intravenous injection of PBS, ICG, or 17-AAG–MnO_2_PEG. Representative images from 3 independent mice per group at each time point (*n* = 3). (B) Ex vivo fluorescence imaging of major organs in mice after 72 h of treatment. (C) Quantitative analysis of mean fluorescence intensity (MFI) in each tissue. *n* = 3, ***P < 0.001.

To further validate the tumor-specific targeting of 17-AAG–MnO_2_PEG, major organs (tumor, heart, liver, spleen, lungs, and kidneys) were excised for fluorescence imaging at 72 h post-treatment (Fig. 5B). Compared to the ICG group, the 17-AAG–MnO_2_PEG group demonstrated stronger fluorescence in tumor tissues, while signals in other major organs, particularly the liver and spleen, were markedly lower, indicating excellent tumor selectivity and minimal nonspecific accumulation in healthy tissues. Quantitative analysis of the mean fluorescence intensity (MFI, Fig. 5C) across tissues revealed that the 17-AAG–MnO_2_PEG group exhibited a markedly higher fluorescence intensity in tumor tissues compared to the ICG group (*P* < 0.001), while the fluorescence intensities in organs such as the liver and spleen were markedly reduced. These findings suggest that 17-AAG–MnO_2_PEG, with its enhanced tumor-targeting properties, can be preferentially enriched in tumor tissues, substantially minimizing nonspecific effects on healthy organs. This selective targeting reduces systemic toxicity, thereby improving the safety and therapeutic efficacy of MWA therapy. It also facilitates the clearance of residual tumor debris by the nanosystem after MWA, thereby enhancing the local therapeutic effect.

### 17-AAG–MnO_2_PEG enables microwave-enhanced synergistic tumor ablation and thermosensitization

In order to validate the in vivo antitumor efficacy of the 17-AAG–MnO_2_PEG nanosystem and to further corroborate the thermosensitization and HSP suppression observed in vitro, tumor-bearing mice were randomly divided into 5 groups with comparable tumor sizes and growth rates (Fig. 6A and Fig. S8). Inclusion of a standalone 17-AAG treatment group enabled assessment of the independent contribution of the HSP90 inhibitor to tumor suppression, thereby distinguishing the effect of drug release from the synergistic interaction between the MnO_2_PEG carrier and MWA. Each group received the designated treatment via intravenous injection, followed by MWA where applicable. Throughout the treatment period, tumor growth, body weight, and overall survival were monitored. All treatment groups exhibited negligible changes in body weight, indicating favorable systemic tolerance and minimal systemic toxicity (Fig. 6B).

**Fig. 6.**
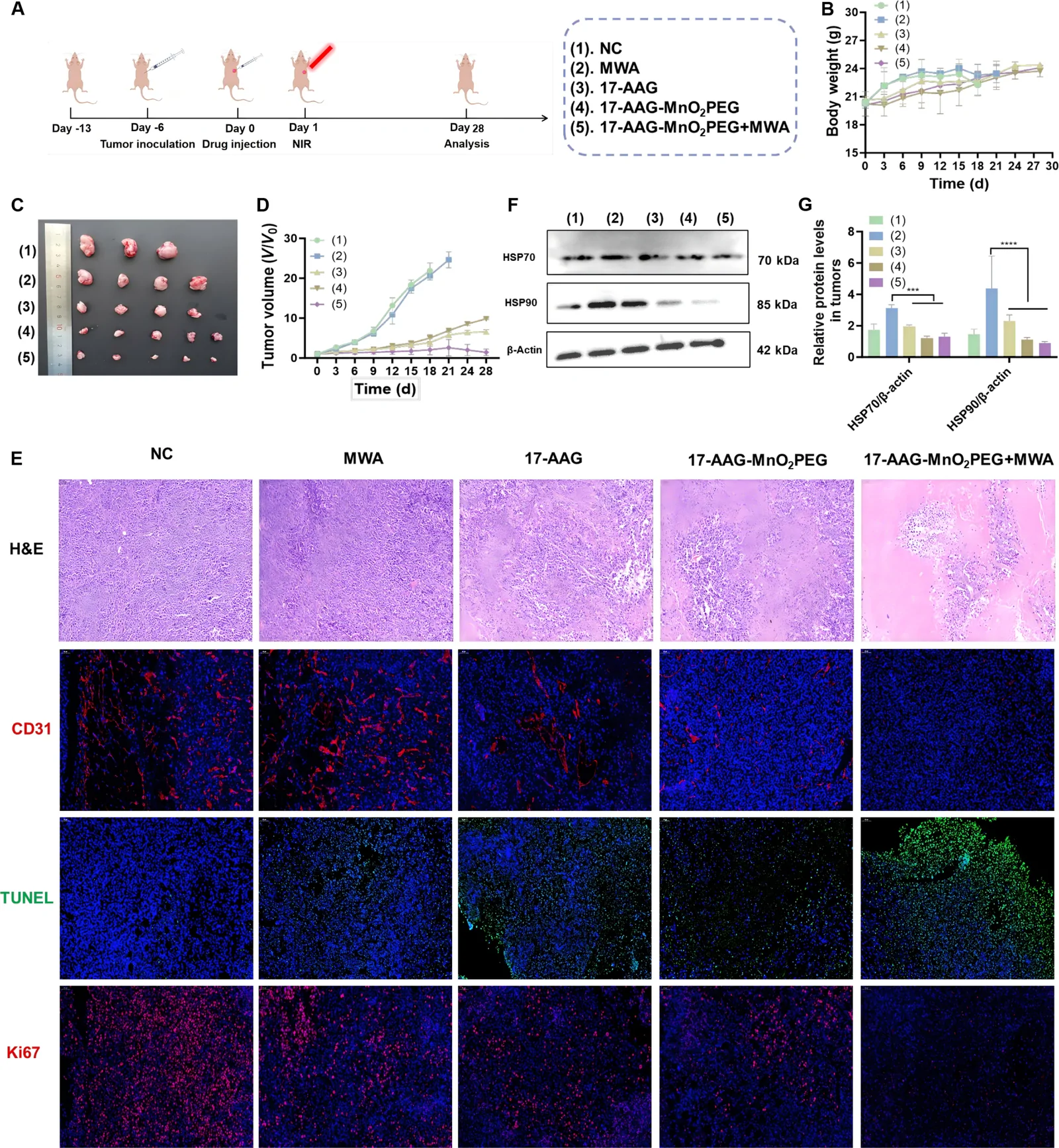
In vivo therapeutic evaluation and mechanistic validation of the 17-AAG–MnO_2_PEG nanosystem. (A) Schematic illustration of the treatment protocol in tumor-bearing mice. (B) Body weight monitoring confirming systemic biosafety. (C and D) Representative tumor images and tumor growth curves. (E) H&E staining and immunofluorescence images of CD31, TUNEL, and Ki67 in tumor tissues. (F and G) Gel images and quantification of HSP70 and HSP90 protein levels. *n* = 3. ***P < 0.001, ****P < 0.0001.

Compared with the control and monotherapy groups, mice treated with 17-AAG–MnO_2_PEG combined with MWA showed the most pronounced inhibition of tumor growth and the highest tumor suppression rate (Fig. 6C and D), demonstrating a markedly enhanced therapeutic efficacy in vivo. Histopathological analysis revealed extensive necrosis and apoptosis within tumor tissues in this group (H&E staining, Fig. 6E). Immunofluorescence analysis further confirmed reduced CD31 expression, increased TUNEL-positive apoptotic cells, and decreased Ki67 levels, indicating suppressed angiogenesis, elevated apoptosis, and reduced proliferative activity within the TME (Fig. 6E).

To elucidate the synergistic mechanism of MWA, the expression of heat shock proteins HSP70 and HSP90 in tumor tissues was assessed. As critical mediators of cellular thermotolerance, HSPs underpin the intrinsic heat resistance of cancer cells; thus, their inhibition by 17-AAG effectively diminishes thermal resilience and amplifies MWA-induced apoptosis. Notably, the 17-AAG–MnO_2_PEG + MWA group exhibited a significant down-regulation of HSP70 and HSP90 compared with the standalone 17-AAG group (Fig. 6F and G), indicating that beyond direct MWA, the nanosystem acts as a thermosensitizer and stress-response suppressor. This synergism effectively converts local thermal stress into irreversible tumor damage, facilitating the clearance of residual tumor debris post-ablation and thereby maximizing local ablation efficacy.

Collectively, these results demonstrate that the 17-AAG–MnO_2_PEG nanosystem integrates tumor-selective drug release, HSP inhibition, and MWA cooperation to achieve efficient and durable tumor suppression in vivo, while the clearance of ablation-induced debris further reinforces local therapeutic outcomes. H&E staining of major organs revealed no significant pathological changes or inflammatory responses in any group, confirming the excellent biocompatibility and minimal off-target toxicity of the nanomedicine (Fig. S9).

Considering the potential neurotoxicity associated with manganese-based nanomaterials, we further evaluated manganese-related brain safety by measuring Mn-SOD activity in brain tissues (Fig. S10). No significant differences in Mn-SOD activity were observed among the treatment groups compared with the PBS group, suggesting negligible Mn accumulation or manganese-associated neurotoxicity under the current treatment regimen.

Afterall, we extended our safety assessment with comprehensive biochemical profiling over a 28-d observation period (Fig. S11), including serum ALT for liver function, ALP for bone/liver function, and CREA and urea (UREA) for renal function. The results revealed no statistically significant abnormalities in any treatment group compared to PBS controls.

### 17-AAG–MnO_2_PEG amplifies MWA-induced antitumor immunity

Building on the mechanistic insights obtained in the MWA-mediated HSP regulation and ICD-driven DC activation by 17-AAG–MnO_2_PEG section—where 17-AAG–MnO_2_PEG was shown to attenuate HSP-mediated thermotolerance, enhance ICD induction, and promote BMDC maturation under MWA—we further investigated whether these immune-priming effects could be sustained in vivo and translated into systemic antitumor immunity. Flow cytometry was performed on splenic T cell populations from tumor-bearing mice subjected to different treatments, with a specific focus on the proportions of CD4^+^ helper T cells and CD8^+^ cytotoxic T cells. Notably, mice receiving 17-AAG–MnO_2_PEG + MWA exhibited a marked increase in both CD4^+^ and CD8^+^ T cell subsets compared with control groups, indicating a more robust systemic immune response and enhanced immunosurveillance potential (Fig. 7A to C).

**Fig. 7.**
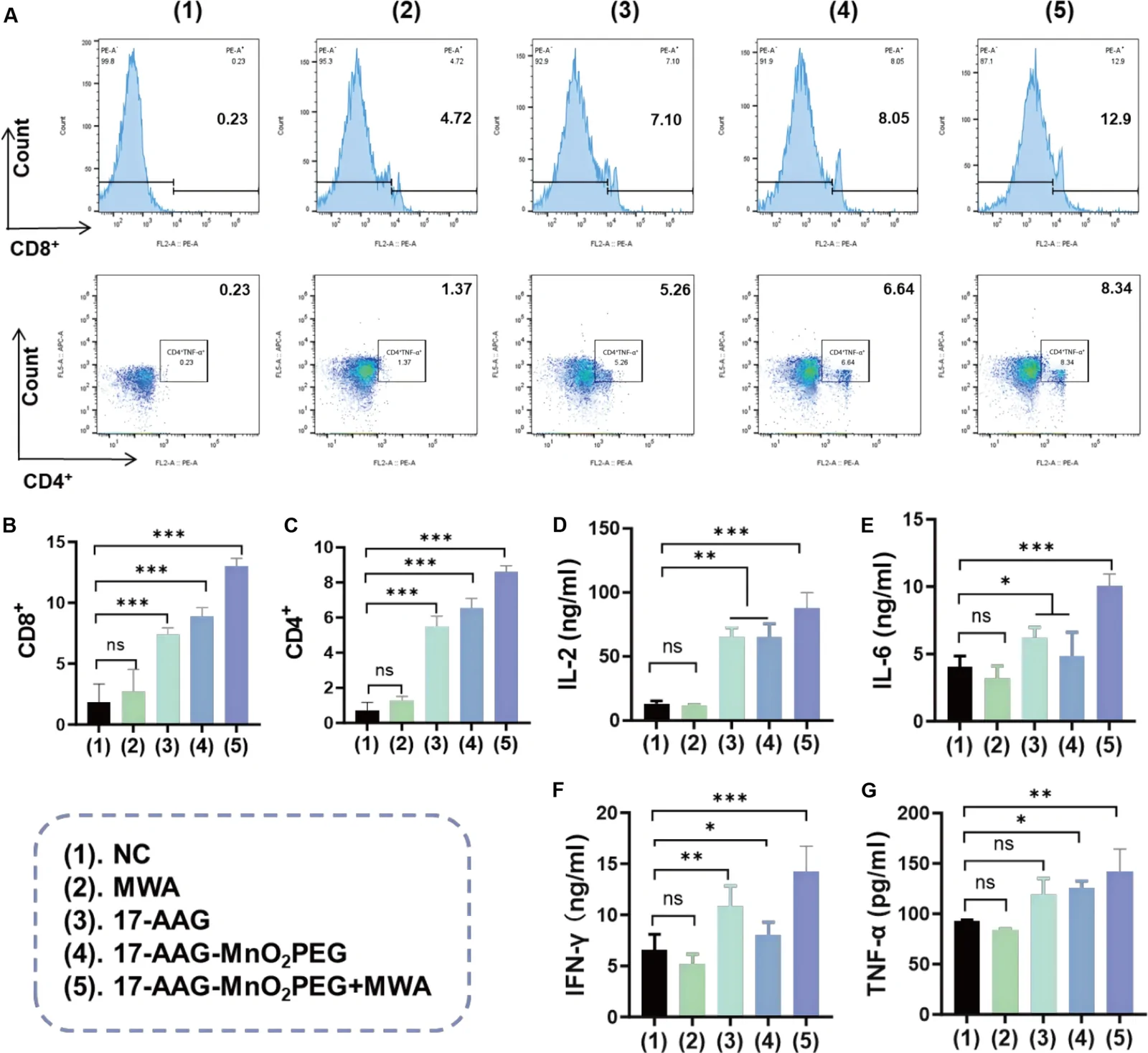
Immunomodulatory effects of 17-AAG–MnO_2_PEG in tumor-bearing mice. (A to C) Flow cytometry analysis of CD4^+^ and CD8^+^ T cell populations in spleens. (D to G) Serum levels of IL-2, IL-6, TNF-α, and IFN-γ measured by ELISA. *n* = 3. Statistical significance: ns = not significant, *P < 0.05, **P < 0.01, ***P < 0.001.

To further validate the immune-activating capacity of 17-AAG–MnO_2_PEG in an immunocompetent setting, splenic lymphocytes isolated from C57BL/6 mice were cocultured with Hepa1-6 hepatoma cells following different treatments. Flow cytometric analysis demonstrated that, compared with the PBS and MnO_2_PEG groups, treatment with 17-AAG–MnO_2_PEG markedly increased the proportions of activated CD4^+^CD25^+^ and CD8^+^CD25^+^ T lymphocytes. Notably, the 17-AAG–MnO_2_PEG + MWA group exhibited the highest percentages of activated CD4^+^ and CD8^+^ T cells among all treatment groups, indicating that MWA further potentiated 17-AAG–MnO_2_PEG-mediated T cell activation (Fig. S12).

To further validate systemic immune activation, serum levels of pro-inflammatory cytokines, including IL-2, IL-6, TNF-α, and IFN-γ, were quantified. The 17-AAG–MnO_2_PEG + MWA group displayed markedly elevated cytokine concentrations (*P* < 0.01), suggesting that local tumor ablation had successfully transitioned into a pro-inflammatory, antitumor immune milieu (Fig. 7D to G). In contrast, MnO_2_PEG or MWA monotherapy failed to trigger significant immune activation (*P* > 0.05), supporting the notion that only through the synergistic inhibition of HSP-mediated thermotolerance and ICD enhancement can MWA achieve effective immunomodulation.

Overall, these findings demonstrate that 17-AAG–MnO_2_PEG can convert MWA-induced tumor cell death into immunogenic signals in vivo, thereby enhancing antigen presentation, promoting T cell priming, and driving the clonal expansion of effector T cells. This process not only contributes to the suppression of residual and recurrent tumors but also may impede metastatic dissemination and support the establishment of durable immunological memory.

In summary, the in vivo results are consistent with the ICD-mediated DC activation observed in the MWA-mediated HSP regulation and ICD-driven DC activation by 17-AAG–MnO_2_PEG section, collectively confirming that 17-AAG–MnO_2_PEG enables MWA to function as both a local cytotoxic modality and an immunotherapeutic intervention, effectively coupling tumor ablation with systemic antitumor immunity

## Discussion and Conclusion

The survival of residual tumor cells and the difficulty in inducing durable antitumor immunity remain key obstacles that fundamentally limit the therapeutic efficacy of MWA in hepatocellular carcinoma and other solid tumors. In this study, we constructed a self-responsive 17-AAG–MnO_2_PEG nanoreactor that harnesses endogenous post-ablation signals—including ROS, cellular debris, and tumor-associated antigens—together with the oxidative microenvironment generated by MWA to achieve site-specific degradation and drug release, thereby fundamentally reprogramming the biological outcomes of incomplete ablation.

The MnO_2_-based nanoreactor locally delivers 17-AAG at the ablation site to suppress HSP90-mediated heat shock responses, diminish thermotolerance, and convert sublethal thermal stress into irreversible proteotoxicity and ICD, resulting in markedly enhanced local tumor eradication. The in situ immune priming further propagates into a robust systemic antitumor response, characterized by DC maturation (CD80/CD86↑), activation of CD4^+^ helper and CD8^+^ cytotoxic T cells, and elevated pro-inflammatory cytokines (IL-2, IL-6, TNF-α, IFN-γ), ultimately leading to suppression of distant tumors and establishment of immune memory. Thus, this strategy successfully transforms local ablation into a body-wide immune event, achieving a cross-scale transition from focal thermal damage to systemic immunosurveillance.

In contrast to conventional adjuvant strategies that rely on exogenous immune agonists or systemic immunotherapy, our approach leverages and amplifies the intrinsic “immune-ignition signals” derived from tumor ablation via a self-feedback nanochemical circuit. This design not only enhances immune stimulation efficiency but also mitigates the risk of systemic immune-related adverse effects. Given that HSP-mediated thermotolerance and oxidative stress are common hallmarks of various solid malignancies, this debris-driven therapeutic paradigm is anticipated to be extensible to other ablation modalities—such as radiofrequency ablation (RFA) and high-intensity focused ultrasound (HIFU)—highlighting its broad translational potential.

Beyond redox modulation, the MnO_2_ component of our nanoreactor is capable of catalyzing H_2_O_2_ to generate O_2_, which may alleviate local tumor hypoxia. Hypoxia is known to stabilize HIF1α, a transcription factor that promotes immunosuppression by up-regulating PD-L1, recruiting myeloid-derived suppressor cells (MDSCs), and impairing effector T cell function. Thus, MnO_2_-mediated O_2_ generation could potentially reverse hypoxia-driven immunosuppression through HIF1α inhibition. Moreover, the released Mn^2+^ ions have been reported to activate the cGAS–STING pathway, a key innate immune signaling axis that promotes type I interferon production and enhances antitumor T cell priming. Collectively, these interconnected pathways—ROS scavenging, hypoxia relief, HIF1α suppression, and STING activation—may synergistically contribute to the robust immune activation observed in our study. However, direct experimental validation of these pathways is warranted in future investigations

Nevertheless, several limitations should be honestly acknowledged. First, we recognize that BALB/c nude mice (T cell-deficient) are not an appropriate model for evaluating T cell-mediated immune responses, and that immunocompetent mice (e.g., C57BL/6) should ideally be used for immunotherapeutic efficacy assessment. While we have provided in vitro evidence using splenic lymphocytes from immunocompetent C57BL/6 mice cocultured with Hepa1-6 cells (Fig. S12), we acknowledge that this does not fully replace an in vivo immunocompetent model, and validation in orthotopic and immunocompetent models remains a top priority for our future studies. Second, although our data suggest the establishment of immune memory (increased CD4^+^/CD8^+^ T cell populations, elevated serum cytokines, and enhanced T cell activation upon restimulation), definitive validation using bilateral tumor models and tumor rechallenge models—which typically require more time—could not be completed and is therefore planned for future investigations. Third, we acknowledge that direct evidence of tumor debris-triggered drug release would further strengthen our claim of a “debris-fueled” mechanism; while our in vitro release assays in H_2_O_2_ (Fig. 2E and F) confirm ROS-responsive release, drug release assays using MWA-treated tumor cell lysates will be pursued in future mechanistic studies. Fourth, while CRT exposure has been confirmed as a key ICD marker (Fig. S5), we acknowledge that HMGB1 release and adenosine triphosphate (ATP) secretion remain to be examined, and comprehensive ICD marker profiling will be included in future work. Fifth, although comprehensive 28-d biochemical profiling (Fig. S11) supports the preliminary safety of our nanosystem, the long-term metabolic fate of the Mn-based nanoreactor, potential off-target effects, interactions with host immune homeostasis, and chronic accumulation risks remain to be fully clarified. Finally, the current use of subcutaneous tumor models facilitates validation of therapeutic feasibility but fails to fully recapitulate the immunological and stromal complexity of orthotopic or metastatic disease; therefore, future studies should employ orthotopic or spontaneous models to better evaluate immune dynamics and therapeutic outcomes, including systematic assessment of anti-metastatic effects. Comprehensive pharmacokinetic profiling, biosafety assessment, and large-animal studies will be required to support clinical translation. Importantly, the parameters used in our MWA system (2.5 GHz, 5 W, 2 min) are clinically relevant and compatible with current clinical MWA protocols for liver cancer, which typically operate at 2.5 GHz with adjustable power and duration. This compatibility facilitates potential clinical translation, as our nanoimmunoreactor could be administered prior to standard MWA procedures without requiring major modifications to existing clinical workflows.

In conclusion, we successfully constructed a 17-AAG–MnO_2_PEG nanoimmunoreactor driven by endogenous immune signals, which achieves synergistic in situ drug release and systemic immune activation by responding to post-ablation tumor debris and the oxidative microenvironment following MWA. Based on the above findings, we draw the following conclusions: 17-AAG–MnO_2_PEG exerts its antitumor effects through a dual mechanism. On one hand, 17-AAG inhibits HSP90-mediated thermotolerance, reducing tumor cell resistance to microwave-induced thermal stress, thereby enhancing MWA ablation efficacy and inducing ICD. On the other hand, MnO_2_ responds to H_2_O_2_ and post-ablation debris in the TME, enabling sustained drug release and modulating local redox balance, which promotes DC maturation and T cell infiltration. More importantly, this strategy converts the otherwise immunosuppressive post-ablation debris into an endogenous immune adjuvant, establishing a self-amplifying immunostimulatory loop that bridges local ablation and systemic antitumor immunity. In summary, this study provides a mechanistically distinct nanoimmunosynergistic strategy to overcome post-MWA tumor recurrence and metastasis, and offers experimental evidence for a tumor debris-driven immunotherapeutic paradigm.

## Data Availability

Data will be made available on request.
