# Supplementary material for: Vulnerability and Immunogenic Cell Death under Microwave Ablation for Synergistic Treatment of Hepatocellular Carcinoma
Source: Biomater Res. 2026 Oct 8;30:0421. doi: 10.34133/bmr.0421 (PMC13646457; doi:10.34133/bmr.0421)
Supplement: Supplementary file 1 [file bmr.0421.f1.docx]

**Supporting Information**

**MnO₂ nanoimmunoreactors potentiate thermal vulnerability and immunogenic cell death under microwave ablation for synergistic treatment of hepatocellular carcinoma**

Zide Chen ^1*^，Hongwei Xu ^2*^， Yufu Peng ^2*^，Mengni Zhu ^4^，Linqing Huang ^5^，Feng Fang ^3#^, Kefei Chen ^2#^

^1^ Department of Interventional Radiology, Guangdong Provincial People's Hospital, Guangdong Academy of Medical Sciences, Southern Medical University, Guangdong, 510080, China.

^2^ Division of Liver Surgery, Department of General Surgery, West China Hospital, Sichuan University, Chengdu 610041, China.

^3^ Department of Hepatobiliary Cancer, Liver cancer research center, Tianjin Medical University Cancer Institute and Hospital, Tianjin, 300060, China.

^4^ Shanghai Key Laboratory of Magnetic Resonance, School of Physics and Electronic Science, East China Normal University, Shanghai, 200241, China.

^5^ The Third Clinical Medical College，Nanjing University of Chinese Medicine，Nanjing, 210023, China.

Author Contributions:

^*^ Zide Chen, Hongwei Xu and Yufu Peng contributed equally to this work.

Corresponding authors:

^#^ Feng Fang (xyfangfeng@aliyun.com), Kefei Chen (Chenkefei_wchscu@163.com)

**Figure S1.** SEM images of MnO₂PEG.

**Figure S2.** UV–Vis absorption spectra of MnO₂ and MnO₂PEG.

**Figure S3.** UV–Vis absorption spectrum of the supernatant after loading 17-AAG onto MnO₂PEG (left), and the corresponding standard calibration curve of 17-AAG (right).

**Figure S4.** Tumor-selective cytotoxicity and cellular uptake of 17-AAG-MnO₂PEG. (A) Cell viability of LO2, HepG2, and SK-Hep-1 cells after different treatments determined by the CCK-8 assay. (B) Quantitative analysis of cellular uptake of R6G-labeled MnO₂PEG and 17-AAG-MnO₂PEG. (C–E) Representative confocal fluorescence images showing the cellular uptake of R6G-labeled MnO₂PEG and 17-AAG-MnO₂PEG in LO2 (C), HepG2 (D), and SK-Hep-1 (E) cells. Red: R6G-labeled nanoparticles; blue: DAPI-stained nuclei. Data are presented as mean ± standard deviation (SD) (n = 3). **p* < 0.05, ***p* < 0.01, ****p* < 0.001.

**Figure S5.** CRT staining of HepG2 and SK-Hep-1 cells after various treatments. DAPI (blue), CRT (green). Scale bar: 100 μm. 17-AAG-MnO₂PEG + MWA showed the strongest CRT signal, indicating potent ICD induction.

**Figure S6.** Representative images of the heart, liver, spleen, lungs, and kidneys collected from mice after administration of different concentrations of the formulations.

**Figure S7.** H&E staining of the heart, liver, spleen, lungs, and kidneys from mice treated with different concentrations of the formulations to assess potential systemic toxicity.

**Figure S8.** Representative images of subcutaneous hepatocellular carcinoma tumors during model establishment (left), body weight changes of mice during the modeling period (middle), and corresponding tumor volume progression over time (right).

**Figure S9.** H&E-stained sections of major organs (heart, liver, spleen, lung, and kidney) from different treatment groups.

**Figure S10.** Mn-SOD activity in brain tissues after different treatments, showing no significant differences among the groups, indicating negligible manganese-associated neurotoxicity.

**Figure S11. Biochemical safety evaluation of 17-AAG–MnO₂PEG treatment in vivo.** Serum biochemical parameters, including total bilirubin (T-Bil), alanine aminotransferase (ALT), alkaline phosphatase (ALP), aspartate aminotransferase (AST), total protein (TP), albumin (ALB), creatinine (CREA), and urea (UREA), were measured after different treatments to evaluate liver and kidney function. No significant differences were observed among the treatment groups, indicating that 17-AAG–MnO₂PEG combined with microwave ablation (MWA) did not induce detectable hepatic or renal toxicity. Data are presented as mean ± standard deviation (SD). ns, not significant.

**Figure S12.** Representative flow cytometry plots and quantitative analysis of the activation status of C57BL/6 splenic T cells co-cultured with Hepa1-6 cells following different treatments.

**Table S1.** Screening of safe concentration for nanomedicine.
